# Are global definitions enough? Revisiting CDAI and SDAI remission cut-offs in Brazilian Rheumatoid Arthritis patients

**DOI:** 10.1186/s42358-026-00532-4

**Published:** 2026-03-13

**Authors:** Lucas Castro Pires, Alisson Pugliesi, Cleandro Pires de Albuquerque, Vitor Alves Cruz, Manoel Barros Bertolo, Ana Paula Monteiro Gomides Reis, Rina Dalva Neubarth Giorgi, Leticia Rocha Pereira, Sebastião Cezar Radominski, Ivanio Alves Pereira, Maria Fernanda Resende Guimarães, Paulo Louzada Junior, Maria de Fátima Lobato da Cunha Sauma, Karina Rossi Bonfiglioli, Claiton Viegas Brenol, Licia Maria Henrique da Mota, Geraldo da Rocha Castelar Pinheiro

**Affiliations:** 1 Department of Orthopedics, Rheumatology, and Traumatology, Universidade Estadual de Campinas (UNICAMP), R. Tessália Vieira de Camargo, 126 - Cidade Universitária, Campinas, SP CEP: 13083-887 Brazil; 2 Department of Rheumatology, Universidade de Brasília, Brasília, Brazil; 3 Department of Rheumatology, Universidade Federal de Goiás, Goiania, Brazil; 4 Department of Rheumatology, Centro Universitário de Brasília (UniCEUB), Brasília, Brazil; 5 Department of Rheumatology, Hospital do Servidor Público Estadual de São Paulo (IASMPE), São Paulo, Brazil; 6 Department of Social Medicine, Universidade do Estado do Rio de Janeiro, Rio de Janeiro, Brazil; 7 Department of Rheumatology, Universidade Federal do Paraná, Curitiba, Brazil; 8 Department of Rheumatology, Universidade Federal de Santa Catarina, Florianópolis, Brazil; 9 Department of Rheumatology, Universidade Federal de Minas Gerais, Belo Horizonte, Brazil; 10 Department of Rheumatology, Universidade de São Paulo, Ribeirão Preto, Brazil; 11 Department of Rheumatology, Universidade Federal do Pará, Belém, Brazil; 12 Department of Rheumatology, Universidade de São Paulo, São Paulo, Brazil; 13 Department of Rheumatology, Universidade Federal do Rio Grande do Sul, Porto Alegre, Brazil; 14 Department of Rheumatology, Universidade do Estado do Rio de Janeiro, Rio de Janeiro, Brazil

**Keywords:** Arthritis, Rheumatoid, Threshold value, Disease activity, Public health

## Abstract

**Background:**

Rheumatoid arthritis (RA) is a chronic systemic inflammatory disease in which achieving remission is the most effective strategy to prevent progression and optimize long-term outcomes. The performance of commonly used disease activity indices has not been well validated in the Brazilian RA population. This study aimed to evaluate the agreement between CDAI/SDAI and the revised Boolean 2.0 remission criteria, which served as the reference standard, and to identify the most accurate CDAI and SDAI remission cut-offs in this population.

**Methods:**

We conducted a cross-sectional analysis of baseline data from a Brazilian Cohort study, which included 840 patients from 11 public hospitals in Brazil. Disease activity was assessed using DAS28-CRP, DAS28-ESR, SDAI, CDAI, and Boolean 1.0/2.0. Agreement was assessed using Cohen’s kappa, and optimal remission cut-offs were determined through ROC curve analysis.

**Results:**

The study population was predominantly female (89.8%), with a mean age of 57 years and a median disease duration of 12 years. DAS28-CRP showed the highest remission rate (39.2%), whereas Boolean 1.0 showed the lowest (15.1%). Strong agreement was found between Boolean 2.0, and both the SDAI (κ = 0.775) and CDAI (κ = 0.692). ROC analysis revealed that the most accurate remission cut-offs were SDAI ≤ 4.3 and CDAI ≤ 3.9, which increased remission detection by 5.9% and 6.2%, respectively.

**Conclusion:**

In our cohort, SDAI ≤ 4.3 and CDAI ≤ 3.9 were the values most closely aligned with Boolean 2.0 remission. These adjusted cut-offs may help minimize overtreatment in resource-limited settings. Prospective studies assessing function, radiographic progression, and quality of life are warranted to confirm their validity in the Brazilian population.

## Background

Rheumatoid arthritis (RA) is a chronic systemic inflammatory disorder characterized by progressive joint destruction, resulting in impaired physical function, work disability, reduced quality of life, and diminished social participation, factors that collectively contribute to a significant global public health burden [1, 2]. Achieving remission has therefore become a central goal in RA management [3–6], as it is associated with better functional outcomes [7–9] and reduced radiographic progression [7, 9, 10].

Over the years, remission criteria have evolved to minimize the risks of both overtreatment and undertreatment [10, 11]. Among the earliest instruments, the DAS28-ESR was developed in the 1990s based on expert clinical assessment and Erythrocyte sedimentation rate (ESR) [12], and later validated using C-reactive protein (CRP) as an alternative biomarker [13]. In 2003, Smolen et al. introduced the Simplified Disease Activity Index (SDAI) [14], followed by the development of the Clinical Disease Activity Index (CDAI) in 2005 by Aletaha et al., which omits laboratory measures for ease of use [15]. The ACR/EULAR Boolean Remission Criteria were first published in 2011 [16], and later revised in 2022 into the more permissive Boolean 2.0 version. This updated version preserves strong predictive validity for both functional and structural outcomes [17] and is currently the recommended remission criterion in the latest EULAR guidelines [5].

Most remission indices incorporate patient-reported outcomes, such as pain and global assessment, which are inherently subjective and can be influenced by cultural and ethnic contexts [18, 19]. Several studies support the use of population-specific remission thresholds [20–24]. In Brazil, where CDAI and SDAI are commonly used for their practicality, adjusting their cut-off values to reflect the local population may improve diagnostic accuracy. To the best of our knowledge, no previous study has proposed alternative cut-offs in the Brazilian context.

This study aimed to evaluate the agreement between commonly used RA activity indices - particularly CDAI and SDAI - and the Boolean 2.0 remission criteria, and to identify optimal remission thresholds for Brazilian RA patients based on data from a large, multicenter real-world cohort.

## Methods

### Study design and population

This study is a cross-sectional analysis of baseline data from a Brazilian Cohort study, which included 840 RA patients from 11 public hospitals in Brazil. This study aimed to characterize the sociodemographic, clinical, and therapeutic profiles of Brazilian RA patients. Details of the cohort design have been previously published [25–27].

The study was approved by the National Research Ethics Committee of the Ministry of Health (number 45781015810015259). Each center also obtained approval from their respective Research Ethics Committees. All participants provided written informed consent.

### Clinical and laboratory assessments

#### Disease activity was assessed using the following indices

- DAS28-CRP: SJC28, TJC28, CRP (mg/L), and PGA (Visual Analog Scale (VAS) 0–10 cm) were calculated using the following formula: DAS28(CRP) = 0.56 × √(TJC28) + 0.28 × √(SJC28) + 0.36 × ln(CRP + 1) + 0.14 × PGA + 0.96. Remission was defined as values ≤ 2.6 [11].
- DAS28-ESR: SJC28, TJC28, ESR (mm), and PGA (VAS 0–10 cm) 0.56 × √(28TJC) + 0.28 × √(28SJC) + 0.70 × ln(ESR) + 0.14 × GH. Remission was defined as values ≤ 2.6 [11].
- SDAI: linear sum of the following variables: SJC28, TJC28, PGA (VAS 0–10 cm), Physician’s Global Assessment (PhGA) (VAS 0–10 cm), and CRP (mg/dL). Remission was defined as values ≤ 3.3 [11].
- CDAI: linear sum similar to SDAI, but does not have the laboratory measurement of CRP (SJC28, TJC28, PGA (VAS 0–10 cm) and PhGA (VAS 0–10 cm)), making it more easily obtained. Remission was defined as CDAI ≤ 2.8 [11].
- Boolean 1.0: comprises four variables and is defined as TJC28 ≤ 1, SJC28 ≤ 1, CRP ≤ 1 mg/dL and PGA (VAS 0–10 cm) ≤ 1 [16].
- Boolean 2.0: similar to Boolean 1.0, with permission for VAS ≤ 2 [17].

Patients with missing data for any index were excluded, resulting in a final sample size of 840 participants from the original 1,115 participants.

### Statistical analysis

Descriptive statistics were used to analyze the baseline patient characteristics. For comparative analyses, the Boolean 2.0 were used as the reference standard in accordance with the most recent EULAR recommendations [5], reflecting their predictive performance for functional and radiographic outcomes, with clinically meaningful positive likelihood ratios across different clinical scenarios, including established disease [17]. The degree of agreement was assessed using Cohen’s κ, and Receiver Operating Characteristic (ROC) curves were used to determine the optimal remission thresholds for each index. All analyses were performed using SAS software (version 9.4).

## Results

### Patient and Rheumatoid Arthritis characteristics

Of the 840 RA patients included, 89.8% were female and 61% were Caucasian. The mean age was 57 years (SD ± 11), and the median disease duration was 12 years (IQR 6.8–19.3). Half (50.6%) of the patients presented with erosive disease at the baseline. Rheumatoid Factor was positive in 78.1% of patients. The most frequently used treatments were Methotrexate (68.1%), corticosteroids (44.5%), and biologic DMARDs (37.7%) (Table 1).

**Table 1:** The participants characteristics

Characteristic	*n*
**Age**, years (mean ± SD)	57 (± 11)
**Female sex** (%)	754 (89.8)
**Years of education** (Median; IQR)	8 (4–11)
**Ethnicity**	
White (%)	512 (61)
Nonwhite (%)	328 (39)
**Disease duration**, months (Median; IQR)	147 (81–231)
**Rheumatoid Factor Positive** (%)	649 (78.1)
**Erosive Disease** (%)	418 (50.6)
**Extra-articular manifestations**	
Subcutaneous Nodules (%)	61 (7.3)
Pulmonary Fibrosis (%)	33 (3.9)
Scleritis (%)	5 (0.6)
Episcleritis (%)	4 (0.5)
**Treatment**	
Corticosteroid (%)	374 (44.5)
Methotrexate (%)	572 (68.1)
Biologic DMARDs (%)	317 (37.7)

Abbreviations: SD, Standard Deviation; IQR, Interquartile Range

### Disease activity scores

Remission rates varied by index: DAS28-CRP showed the highest remission rate (39.2%), while Boolean 1.0 showed the lowest (15.1%). Approximately 20% of patients met CDAI or SDAI remission criteria (Table 2).

**Table 2:** Remission rates according to different indices

	Median (IQR)	Reference value for disease remission	Remission Rate (%)
DAS28-CRP	3.0 (2.2–4.2)	≤ 2.6	329 (39.2)
DAS28-ESR	3.5 (2.5–4.6)	≤ 2.6	227 (27.0)
CDAI	9 (3.4–18.3)	≤ 2.8	173 (20.6)
SDAI	10.4 (4.3–20.1)	≤ 3.3	161 (19.2)
Boolean 1.0	-	-	127 (15.1)
Boolean 2.0	-	-	164 (19.5)

Abbreviations: IQR, Interquartile Range

### Agreement with Boolean 2.0

**Fig. 1:**
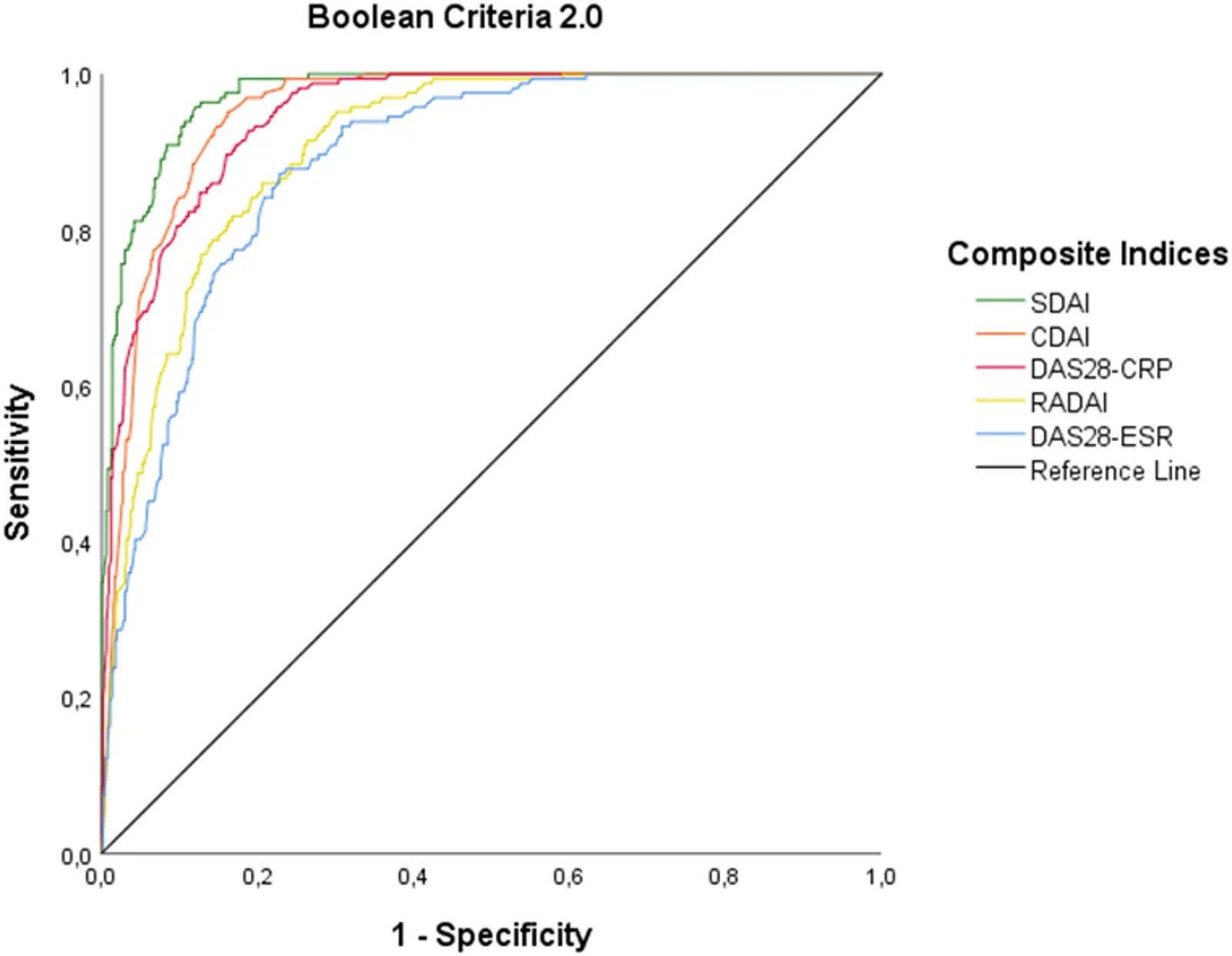
ROC curve illustrating the sensitivity and 1-specificity of the different metrics in relation to CB 2.0

The Boolean 2.0 criteria demonstrated strong agreement with SDAI (κ = 0.775) and CDAI (κ = 0.692) and moderate agreement with DAS28-ESR (κ = 0.527) and DAS28-CRP (κ = 0.525). ROC curve analysis (Fig. 1) demonstrated the excellent performance of SDAI and CDAI in identifying remission per Boolean 2.0.

- SDAI AUC: 0.973 (95% CI: 0.964–0.983).
- CDAI AUC: 0.951 (95% CI: 0.937–0.964).
- DAS28-CRP AUC: 0.947 (95% CI: 0.933–0.962).
- DAS28-ESR AUC: 0.888 (95% CI: 0.864–0.912).

### Proposed cut-off points

**Fig. 2:**
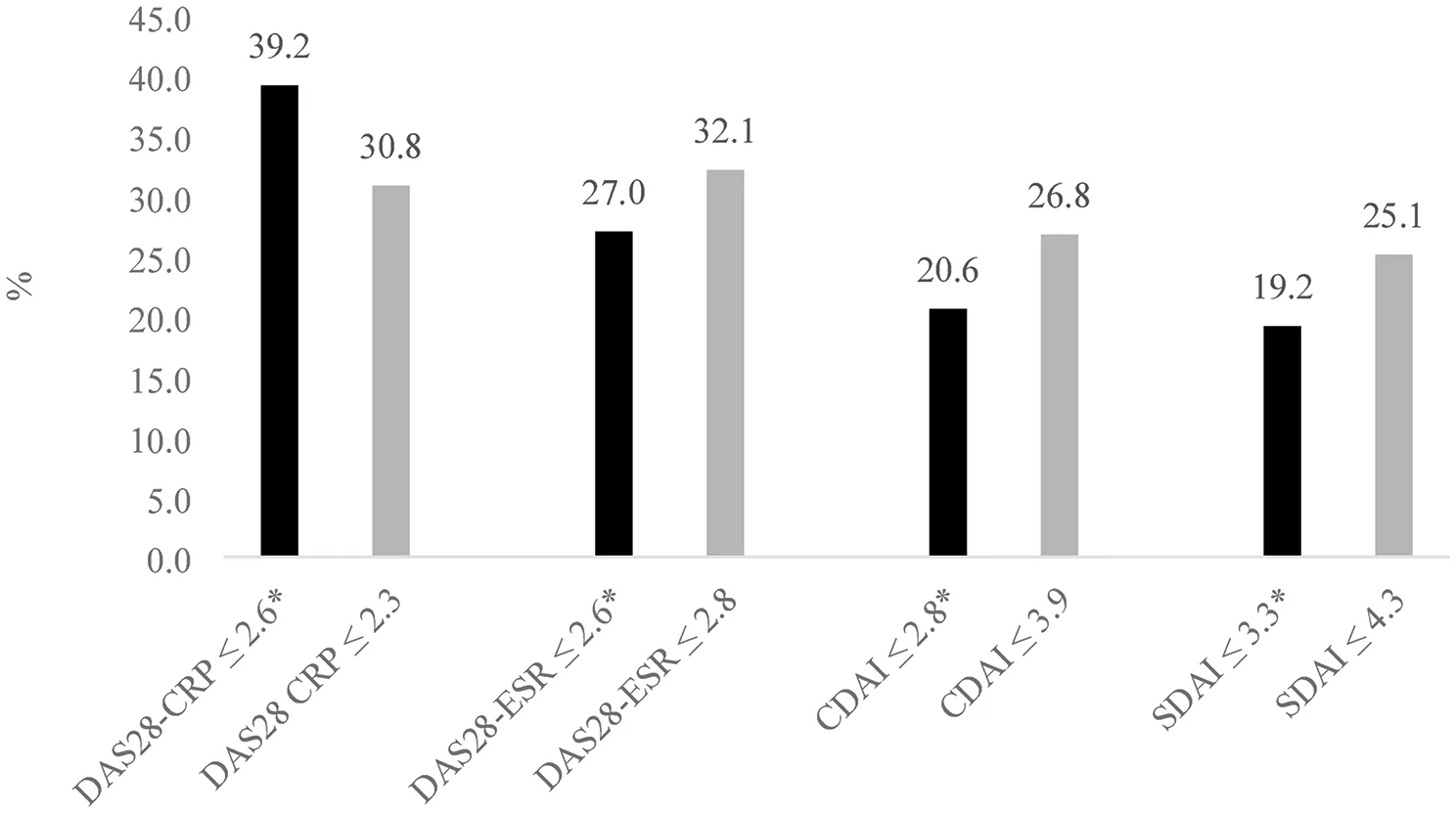
Remission rates according to DAS28, SDAI, and CDAI Cut-Offs. *Current reference value

The most accurate remission thresholds were SDAI ≤ 4.3 and CDAI ≤ 3.9, both of which outperformed the conventional cut-offs. These adjusted thresholds increased the remission detection rates by 5.9% and 6.2%, respectively (Fig. 2; Table 3).

**Table 3:** Sensitivity and specificity of proposed cut-off points for remission

Metric	Cut-off	Sensitivity (%)	Specificity (%)
DAS28-CRP	≤ 2.6*	97.6	75.0
	≤ 2.3	86.0	85.9
DAS28-ESR	≤ 2.6*	75.6	84.8
	≤ 2.8	81.7	79.9
CDAI	≤ 2.8*	77.4	93.2
	≤ 3.9	88.4	88.3
SDAI	≤ 3.3*	81.1	95.9
	≤ 4.3	90.9	91.7

*Current reference value

## Discussion

This study confirmed the strong concordance between Boolean 2.0 and the SDAI and CDAI indices, reinforcing previous validation efforts [17] and highlighting the structural and conceptual similarities among these indices. Notably, SDAI ≤ 4.3 and CDAI ≤ 3.9 showed the highest accuracy in identifying remission compared with the Boolean 2.0 criteria in the Brazilian RA population.

The CDAI offers a practical advantage in resource-limited settings because it does not require laboratory data. Its relevance is supported by its correlation with functional, radiographic, and ultrasound outcomes [10, 15, 28], and its use in monitoring IL-6 inhibitor therapies [29].

Although the increase in remission rates using the new cut-offs was modest, these findings have important implications for minimizing overtreatment in a disease that affects over one million Brazilians [30, 31].

As expected, DAS28-based scores demonstrated lower concordance with Boolean 2.0, aligning with literature suggesting that these indices may overestimate remission due to residual disease activity [28, 32, 33], even when stricter cut-off points are applied [34]. This limitation reflects the intrinsic influence of the mathematical weighting of the DAS28 components on the overall score [34, 35].

Remission rates in this cohort were lower than in international studies [36–40], possibly due to a real-world study design, longer disease duration, higher rates of seropositivity and erosive disease, and limited access to advanced therapies.

This is the first large-scale study to evaluate Boolean 2.0 in Brazilian RA patients, providing crucial insights for both clinical care and health policy.

Our findings should be interpreted in light of certain limitations. We were unable to assess radiographic progression or functional outcomes at different cut-offs, and the use of advanced therapy was largely restricted to TNF inhibitors. With the broader availability of other biologic DMARDs, remission rates are expected to rise. In addition, we did not evaluate the presence or impact of concomitant fibromyalgia on the performance of the remission metrics. Finally, we focused exclusively on remission cut-offs; further studies are needed to refine other key treat-to-target classifications, such as Low Disease Activity, particularly in patients with established disease.

## Conclusion

Our findings suggest that the traditional SDAI and CDAI cutoff points may underestimate remission in Brazilian patients. Revised thresholds of SDAI ≤ 4.3 and CDAI ≤ 3.9 showed greater accuracy when compared with the Boolean 2.0 reference. Prospective studies assessing function, radiographic progression, and quality of life are needed to validate these proposed adjustments.

## Data Availability

The datasets used and/or analyzed during the current study are available from the corresponding author upon reasonable request.
